# The Plasticity of CD4^+^CD25^+^FOXP3^+^CD127^low^ T Cells in Patients with Metastatic Renal Cell Carcinoma in the Course of Interferon-Alpha Immunotherapy

**DOI:** 10.1155/2018/7828735

**Published:** 2018-05-02

**Authors:** Maria S. Sayapina, Svetlana N. Bykovskaia

**Affiliations:** ^1^N.N. Blokhin Russian Cancer Research Center, Ministry of Health of Russia, 23 Kashirskoe Shosse, Moscow 115478, Russia; ^2^Pirogov Russian National Research Medical University, 1 Ostrovityanova St., Moscow 117997, Russia; ^3^Regenex LLC, Skolkovo Innovation Center, Moscow, Russia

## Abstract

**Aims:**

To examine changes in subpopulation of CD4^+^CD25^+^Foxp3^+^CD127^low^ T lymphocytes (Treg) and their association with the efficiency of the IFN-*α* therapy.

**Materials and Methods:**

Pts with mRCC who had undergone nephrectomy were treated with IFN-*α* at a dose of 6 × 10^6^ U/day three times a week (*n* = 18). An immunophenotypic analysis of lymphocytes in peripheral blood expressing CD4, CD25, CD127, and Foxp3 antigens could be performed in 18 pts before, 2 weeks, and 2 mo after IFN-*α* therapy and 22 normal volunteers. Blood samples were collected at baseline and 2 mo after treatment start. Serum levels of TGF-*β*1, IL-17A, and Epo were measured by ELISA.

**Results:**

PR was achieved in 3 (16.6%) pts who received first-line therapy. Long-lasting SD (≥6 months) was noted in 6 (33.3%) pts. The median progression free survival (PFS) was 4 mo (95% CI: 2-NE). The study of the population of Treg indicated that there were no significant differences in the groups depending on the effect (*p* = 0.71). In one patient, the reduction of Treg cells was associated with increased TGF-*β* and IL-17 levels, whereas in other two pts the increase in Treg cells was associated with decreased TGF-*β* and IL-17 levels. The endogenous levels of Epo did not show significant correlation with response to IFN-*α* immunotherapy. In the patient subgroup with an initial value of MCH > 31 pg, the median PFS was not achieved, but in the subgroup with an initial value of MCH < 31 pg, the median PFS was 2 months (*p* = 0.032).

**Conclusions:**

In our study, we have described functional plasticity of Treg cells, which prevents them from being used as a prognostic marker. The conversion of Treg cells into Th17 can serve as a basis for the development of a new specific immunotherapeutic method in oncology after confirmation in the experiment in vitro. Given the small dataset, the results will need further validation.

## 1. Introduction

Naive CD4^+^ cells differentiate into T helper (Th1, Th2, Th9, and Th17) and regulatory T (Treg) cells to execute their functional activities. Th1, Th2, and Th17 cells play an important role in the immune response against intracellular pathogens and extracellular parasites. Nevertheless, immune responses exerted by these T helper cells also cause autoimmune and inflammatory diseases. FOXP3^+^ Treg cells play a key role in inducing immune tolerance and in the limitation of the inflammatory response executed by Т helper cells. Although Treg and Th17 cells have different functions, the differentiation of both cell subsets depends on TGF-*β* [1–3]. Although Th1 and Th2 subsets are considered as definitive and mutually exclusive lineages, it seems that Th17 and Treg subsets do not represent stable differentiation processes and retain plasticity allowing them to adapt to different environments. Th17 cells are associated with inflammatory and autoimmune diseases in mice and human. Notably, antigen-specific Th17 cells and their related cytokines are highly pathogenic and exhibit detrimental roles in multiple sclerosis, psoriasis, systemic lupus erythematosus, rheumatoid arthritis, inflammatory bowel disease, and asthma [3–6]. While Th17 cells function as pathogenic Th cells in autoimmunity, their role in cancer is still under debate. In addition, whether Th17 plasticity and conversion into several Th cells will, as described in many inflammatory diseases, similarly happen in tumor context will be discussed in our study [3]. This study shows the influence of IFN-*α* immunotherapy on Treg cells in patients with metastatic renal cell carcinoma (mRCC). IFN-*α* can affect tumor cell functions by multiple mechanisms. Of note, several studies have then underscored new immunomodulatory effects of IFN-*α*, including activities on T cells and dendritic cells, which may lead to IFN-induced antitumor immunity. In addition, recent reports on new immune correlates in cancer patients responding to IFN-*α* represent additional evidence on the importance of the interactions of IFN-*α* with the immune system for the generation of a durable antitumor response. Second, there is IFN-*α*-mediated lineage-specific regulation of MHCII genes expression. On the whole, the data suggest an upregulated renal immune microenvironment including Treg cells in the presence of IFN-*α* [7–9].

Previous studies have demonstrated that, under hypoxic conditions, CD4^+^ cells preferentially differentiate into Th17 rather than Treg cells due to HIF 1-*α* accumulation with subsequent transcriptional activation of Th17-associated ROR*γ*t gene and promotion of proteasomal degradation of Foxp3 gene [10]. Moreover, several genes (VEGF, PDGF, TGF-*α*, and Epo) which are involved in hypoxia adaptation and regulate angiogenic processes are activated. Erythropoietin (Epo) is the only hematopoietic growth factor whose production is regulated by local hypoxia [11, 12]. Although less than 5% of renal cell tumors produce erythropoietin, its production may identify a subset of individuals with renal cell cancer responsive to IL-2 and IFN-*α* [13]. So this study also may represent a subset of patients that would benefit from interferon-alpha (IFN-*α*) treatment.

## 2. Materials and Methods

18 patients with mRCC, who had undergone nephrectomy, were treated with subcutaneous IFN-*α* at a dose of 6 × 10^6^ U/day three times a week. General characteristics of patients are shown in Table 1. First-line therapy was given to 16 patients and second-line therapy to 2 patients. The patients were stratified according to the MSKCC criteria. Histological diagnosis was clear cell variant of RCC in all the patients. The median age was 55 years (41–77).

The patients from Russia were selected for additional analysis of immune response during IFN-*α* immunotherapy and study the influence of IFN-*α* immunotherapy on Treg cells. To evaluate the clinical effectiveness of therapy, the Response Evaluation Criteria in Solid Tumors (RECIST) were used. Follow-up examinations (CT, US) were performed every 2 months and also when clinical signs of disease progression appear.

This study was carried out in accordance with the International Conference on Harmonisation of Technical Requirements for Registration of Pharmaceuticals for Human Use Good Clinical Practice guidelines and the ethical principles outlined in the Declaration of Helsinki. The study protocol has been reviewed by Local Ethics Committee within Blokhin Cancer Research Center. All patients provided written, informed consent before undergoing any study-related procedures.

### 2.1. Flow Cytometry Analysis

Immunologic parameters were evaluated in the Cell Technology Laboratory of the N.I. Pirogov Russian National Research Medical University 1 week before, 2 weeks, and 8 weeks after immunotherapy during control examinations. The expression of superficial markers was evaluated according to the protocol developed by Miltenyi Biotec GmbH, Germany (manufacturer of antibody kits). The kit contained the following antibodies: FITC-labeled CD3, clone SK7; PE-labeled CD8, clone SK1; CD45 labeled with PerCP, clone 2D1 (HLe-1); APC-labeled CD4, clone SK3; PE-labeled CD16, clone B73.1; PE-labeled CD56, clone NCAM 16.2; and APC-labeled CD19, clone SJ25C1. For intracellular staining of lymphocytes by FoxP3 antibodies, the Treg Detection Kit (Miltenyi Biotec GmbH, Germany) was used. Measurements were performed using a flow cytometer (Miltenyi Biotec GmbH, Germany). The percentage of cells positive for specific markers was calculated using the MACSQuantify (TM) Software (Miltenyi Biotec GmbH, Germany).

### 2.2. Statistical Analysis

The statistical analysis and data processing were done using the program STATISTICA 13 and describe.png. When comparing immunologic parameters, the Wilcoxon signed-rank test and* t*-test as well as Spearman and Pearson criteria were used. Differences were considered significant at *p* < 0.05 (two-sided *p* value). The time to disease progression was determined by the Kaplan-Meier method from the start date of immunotherapy to the start date of disease progression or death of any cause.

### 2.3. Enzyme-Linked Immunosorbent Assay (ELISA)

Immunoenzymatic assays were performed in the Laboratory of Biochemistry of the N.N. Blokhin Russian Oncological Research Center and in the Department of Laboratory Diagnostics of the A.F. Tsyb Medical Radiological Research Center. The protein concentration in blood serum obtained by centrifuging blood at 3000 rev/min, 4°С, for 10 minutes (centrifuge PС-6, Russia) was determined before and 2 months after treatment using a standard method. Then, 300–400 mcl of serum was poured into 2 plastic test tubes and stored at −80°С before assaying. Immunoenzymatic assays were performed using standard kits for a direct immunoenzymatic assay according to the manufacturer's instructions (Table 2).

## 3. Results

The efficacy of IFN-alpha immunotherapy was assessed in 18 patients. On beginning IFN-*α* immunotherapy, 16 patients received first-line therapy, and 2 patients received second-line therapy. Partial response was achieved in 3 (16.6%) patients (received first-line therapy). Long-lasting stable disease (≥6 months) was noted in 6 (33.3%) patients including 2 patients who received second-line therapy. The overall disease control rate (partial remission + long-lasting stable disease) was 50%. One (5.5%) patient showed a delayed partial response after disease progression. On beginning immunotherapy, all the patients had metastases in different organs.

The most common site where cancer spread was the lung (88.8%). In the whole group of 18 patients, the median survival from starting immunotherapy to disease progression was 4 months (95% CI: 2-NE), whereas median PFS (progression-free survival) was 2 months in patients who received first-line therapy (*n* = 16). Median follow-up of surviving patients has been 18 months. These patients continue to be assessed.

*Assay for CD4* ^*+*^ *CD25* ^*+*^ *Foxp3* ^*+*^ *CD127* ^*low*^ *T Cells*. An immunophenotypic analysis of lymphocytes in peripheral blood expressing CD4, CD25, CD127, and Foxp3 antigens could be performed in 18 patients before, 2 weeks, and 2 months after IFN-*α* therapy at the time of the initial evaluation of the efficacy of IFN-*α* therapy.

**Figure 1:**
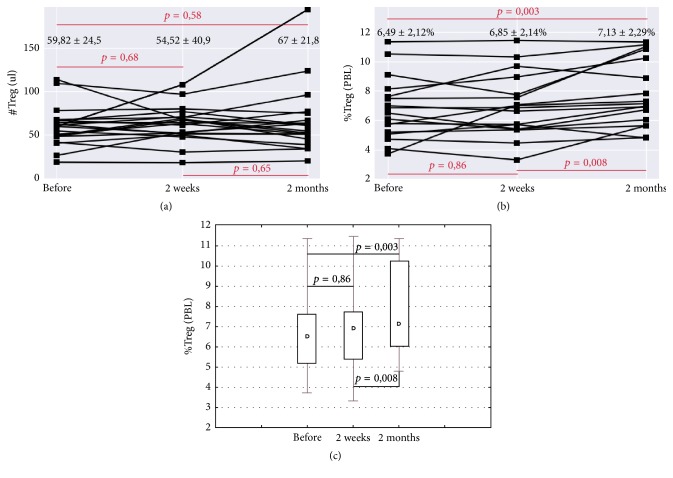
Absolute number (a) and percentage ((b) and (c)) of Treg cells in peripheral blood of patients with mRCC before, 2 weeks, and 2 months after IFN-*α* immunotherapy.

In the first stage, the influence of immunotherapy on CD4^+^CD25^+^Foxp3^+^CD127^low^ T cells in the whole patient group (*n* = 18) was assessed. After 2 weeks there was no significant increase in the population of CD4^+^CD25^+^Foxp3^+^CD127^low^ T cells, while from 2 weeks to 2 months there was a significant increase from 6.85 ± 2.14% to 7.13 ± 2, 29% (*p* = 0.008). It should be noted that, in the analysis of the absolute amount of Treg in 1 *μ*l of blood after 2 weeks, there was a decrease in Treg cells from 59.82 ± 24.5 to 54.52 ± 40.9 at 2 weeks after immunotherapy and then there was an increase up to 67.12 ± 21.8 at 2 months after immunotherapy; however, the differences were unreliable (*p* > 0.05) (Figure 1).

**Figure 2:**
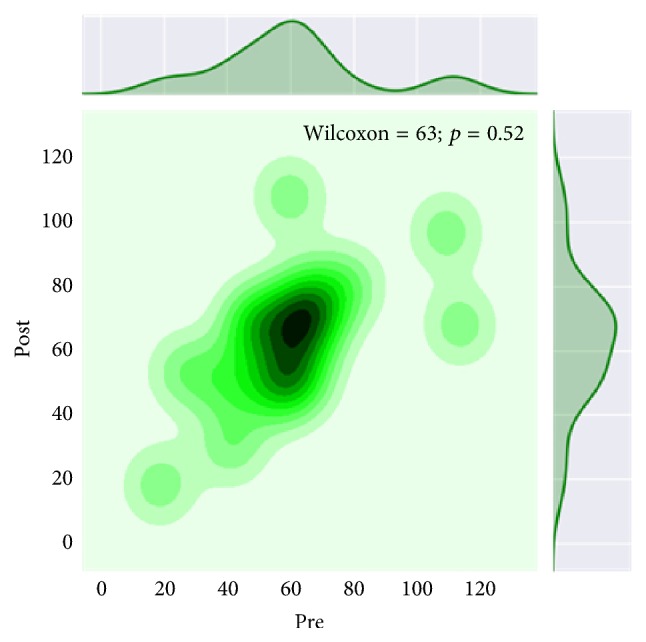
The absolute number of CD4^+^CD25^+^Foxp3^+^CD127^low^ T cells in 1 mcl of peripheral blood in patients with mRCC before and 2 months after immunotherapy.

Taking into account more marked significant changes in the absolute number of Treg cells, the comparison of the absolute numbers between immunological parameters was further conducted before and 2 months after starting therapy (Figure 2).

In the next stage, all the patients were grouped according to response (progression, partial response, and stable disease). Baseline values of the absolute numbers of CD4^+^CD25^+^Foxp3^+^CD127^low^ T cells were compared between groups, and changes of parameters in each group depending on the treatment efficacy were studied.

The study of the population of CD4^+^CD25^+^Foxp3^+^CD127^low^ T cells indicated no significant differences in the groups depending on the effect (*p* = 0.71). Moreover, in patients who experienced early disease progression, the absolute number of CD4^+^CD25^+^Foxp3^+^CD127^low^ T cells in 1 mcl of peripheral blood prior to therapy was lower than in patients who achieved stable disease and partial response: 54.7 ± 18.8 versus 60.4 ± 26.8, 62.4 ± 31.9, respectively.

**Figure 3:**
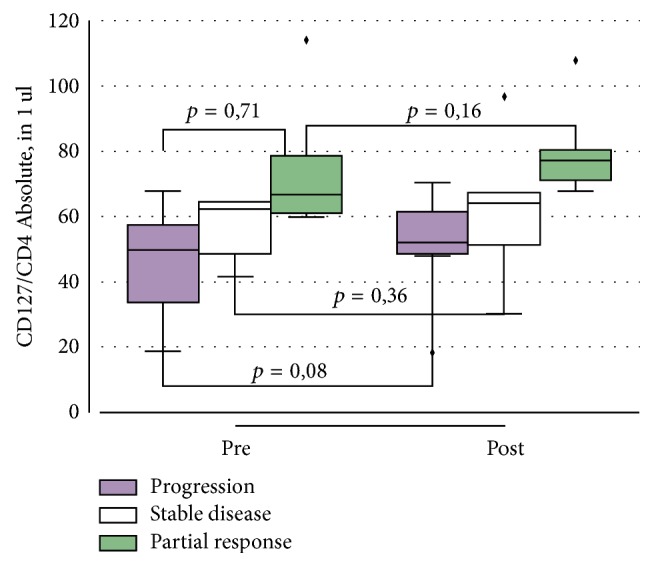
The absolute number of CD4^+^CD25^+^Foxp3^+^CD127^low^ T cells in peripheral blood of patients with mRCC in the presence of disease progression, stable disease, and partial response before and 2 months after IFN-*α* immunotherapy.

Nonetheless, 2 months after starting immunotherapy, this lymphocyte population tended to increase from 54.7 ± 18.8 to 61 ± 12.3 (*p* = 0.08) in early progression group, from 60.4 ± 26.8 to 65.9 ± 28.16 (*p* = 0.36) in stable disease, and from 62.4 ± 31.9 to 67 ± 23.18 (*p* = 0.16) in partial response groups (Figure 3). We evaluated the amount of this cell population in 22 healthy donors; it was 77.12 ± 5.2 cells per mcl of blood.

**Figure 4:**
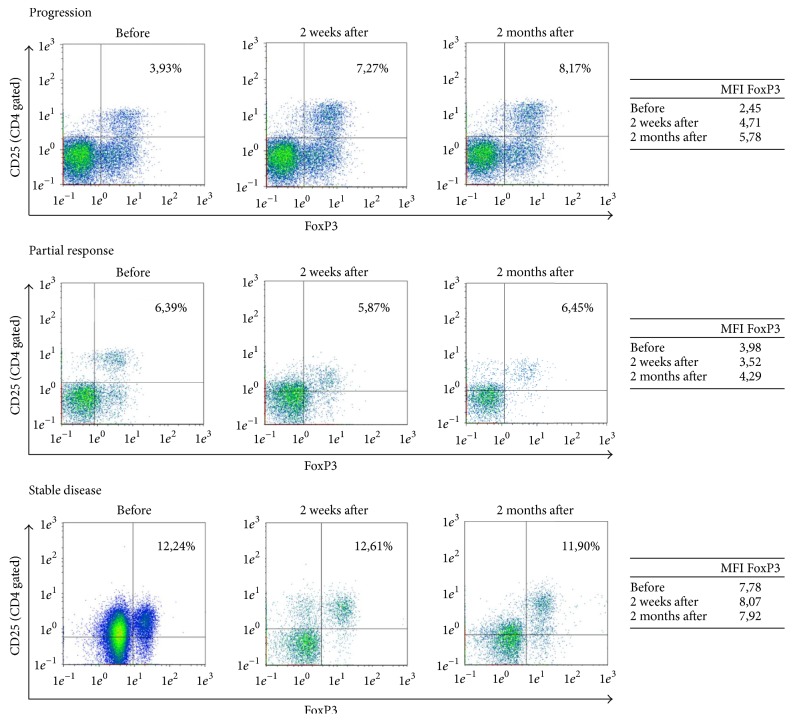
Foxp3 expression in peripheral blood of patients with mRCC in the course of IFN-*α* immunotherapy.

Figure 4 shows Foxp3 expression in peripheral blood of patients with mRCC in dependence of clinical response. The presented data demonstrate correlation between percentage and MFI of Foxp3 expression.

In the next stage, serum factors TGF-*β* and IL-17A were analyzed in 18 patients with mRCC before and 2 months after IFN-*α* immunotherapy. In the course of the treatment, TGF-*β* tended to increase from 11.3 ± 12.4 to 13 ± 10.1 ng/ml (*p* = 0.1), and IL-17A was significantly elevated from 0 ± 4.29 (median + sd) to 0.16 ± 1.7 ng/ml (*p* = 0.003).

**Figure 5:**
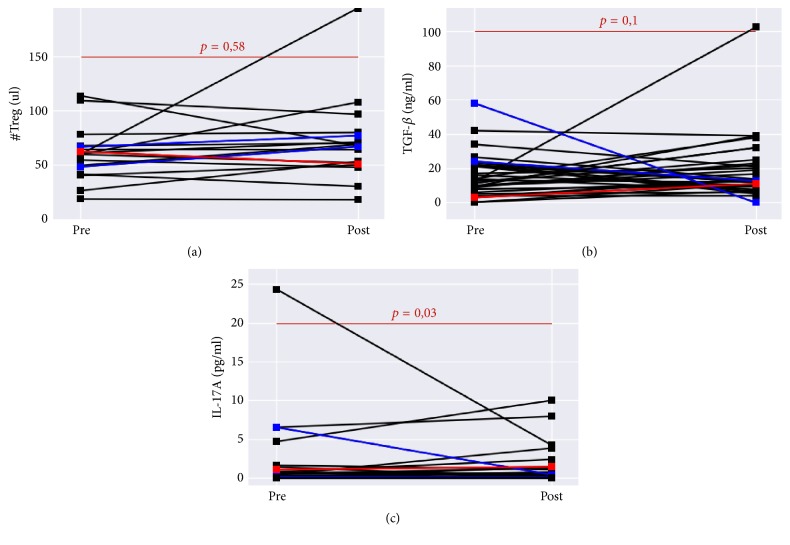
Relationship between the absolute number of Treg cells (a) and serum levels of TGF-*β* (b) and IL-17 (c) in patients with clinical response (blue and red) before and after immunotherapy.

The analysis of the relationship between the level of serum factors and changes of the absolute number of CD4^+^CD25^+^Foxp3^+^CD127^low^ T cells in patients, who had achieved clinical response, revealed that in one patient the reduction of Treg cells was associated with increased TGF-*β* and IL-17 levels, whereas in other two patients the increase in Treg cells was associated with decreased TGF-*β* and IL-17 levels (Figure 5). The obtained data may be indicative of the conversion of Treg cells into Th17 cells (secreting primarily IL-17) and the plasticity of Treg cell function, respectively.

In our study, we measured* the endogenous levels of Epo* in 18 patients with mRCC prior to starting IFN-*α* immunotherapy. The obtained results did not show significant correlation (*p* = 0,8) with response to IFN-*α* immunotherapy because only in one patient (5.5%) the initially elevated endogenous Epo concentration was associated with clinical response.

**Figure 6:**
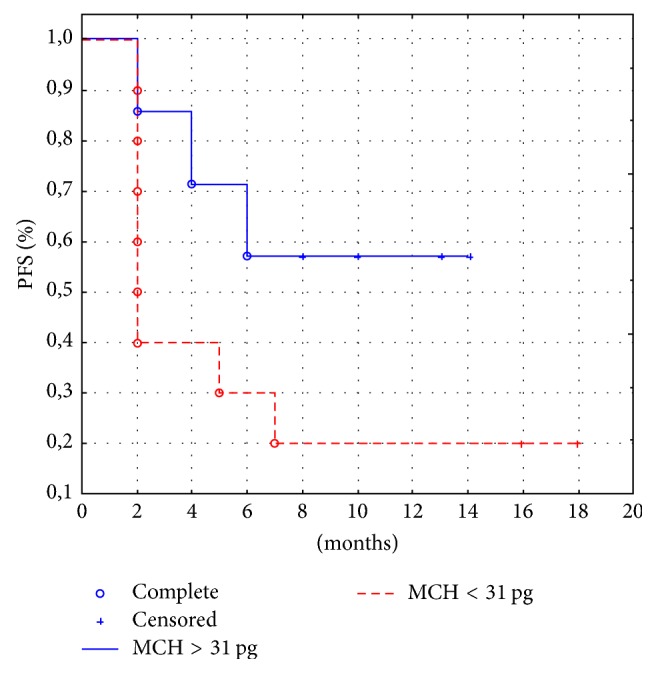
Progression-free survival depending on initial MCHC in peripheral blood (*p* = 0.032).

At the same time, a correlation between* the mean cell hemoglobin (MCH)* and response was found. The effect of initial values of MCH on progression-free survival in 17 from 18 patients with mRCC was analyzed later on. In the patient subgroup with an initial value of MCH > 31 pg, the median progression-free survival was not achieved, but in the subgroup with an initial value of MCH < 31 pg, the median progression-free survival was 2 months (*p* = 0.032) (Figure 6). Nonetheless, clinical response was achieved in 57% of patients with initially elevated MCH (38.8%).

## 4. Discussion

Our study of the population of CD4^+^CD25^+^Foxp3^+^CD127^low^ T cells indicated no significant differences in the groups depending on the effect (*p* = 0.71). The absolute number of CD4^+^CD25^+^Foxp3^+^CD127^low^ T cells in progression group was lower than that in partial response group and stable disease group. But in the latter two groups, it was not higher than in donors. Moreover, in all groups, this cell population tended to grow regardless of response. The obtained results deny the concept that CD4^+^CD25^+^Foxp3^+^CD127^low^ T cells could be used as a negative prognostic marker in oncologic diseases [14]. It is necessary to determine the function of Treg cells taking into account their plasticity.

The comparison of serum concentrations of TGF-*β* and IL-17 with the absolute number of Treg cells enabled to identify three patients who showed clinical response. In one of them, reduction of Treg cells was associated with increased levels of TGF-*β* and IL-17, whereas in other two patients increased numbers of Treg cells were associated with decreased levels of TGF-*β* and IL-17. This made the authors assume that, in the first patient, Treg cells were converted into Th17 cells, which was possible in the presence of anti-inflammatory factors (IL-6) and TGF-*β* [1]. Nowadays, the functional role of Th17 cells in oncology remains controversial [15]. It is known that this cell population prevails in autoimmune diseases in conditions required to suppress the function of Treg cells [2]. Increased numbers of Treg cells in the above-mentioned two patients were associated with reduced TGF-*β* levels and clinical response, which may suggest that initially the function of Treg cells was nonsuppressive.

In previous studies, conversion of Treg into Th17 cells was described in cell cultures using indole-amino-2, 3-dioxygenase (IDO) that stimulated tryptophan catabolism, thereby suppressing T cell immunity [16]. Treg cells could be also converted into Th17 cells under hypoxic conditions [10]. In our study, only one patient showed the initially raised level of endogenous Epo associated with clinical response. Several studies have reported cases of complete response to HD-IL2 and IFN-*α* immunotherapy in patients with mRCC in conditions of erythrocytosis and elevated erythropoietin. More studies are needed to validate this marker.

In such a context, a correlation between the endogenous Epo levels and response was suggested. The obtained results did not show significant correlation with response to IFN-*α* immunotherapy because only in one patient (5.5%) the initially elevated endogenous Epo concentration was associated with clinical response. At the same time, a correlation between the mean cell hemoglobin (MCH) and response was found. The study of the effect of initial values of MCH on progression-free survival revealed that, in the patient subgroup with an initial value of MCH > 31 pg, the median progression-free survival was not achieved, but in the subgroup with an initial value of MCH < 31 pg, the median progression-free survival was 2 months (*p* = 0.032). Thus, MCH can be used as a positive prognostic factor and in some cases as a predictor factor of response to IFN-*α* immunotherapy in patients with mRCC. Similar results have not been previously reported in the literature. But given the small dataset the results will need further validation.

The influence of the tumor microenvironment on T cell plasticity is not fully known. Several immunologic studies demonstrated that Treg cells could be converted into Th17 cells and then into Th1 cells in the presence of IL-1, IL-2, IL-21, and IL-23 [3]. However, this scenario has not been illustrated in oncology. As the human thymus does not contain Treg cells producing IL-17, IL-17+FOXP3+Treg cells are generated in the periphery [17]. IL-6 induces the development of Th17 cells from naive T cells together with TGF-b; in contrast, IL-6 inhibits TGF-b induced Treg differentiation. Dysregulation or overproduction of IL-6 leads to autoimmune diseases such as multiple sclerosis (MS) and rheumatoid arthritis (RA), in which Th17 cells are considered to be the primary cause of pathology. Given the critical role of IL-6 in altering the balance between Treg and Th17 cells, controlling IL-6 activities is potentially an effective approach in the treatment of various types of cancer [18–23].

Thus, for the first time, we observed the plasticity of immunoregulatory of CD4^+^CD25^+^Foxp3^+^CD127^low^ T cells against the background of immunotherapy of INF-*α* in the patient with mRCC. The conversion Treg cells into Th17 after confirmation in the experiment in vitro in the long term can be considered as a basis for the development of a new drug for cancer, including mRCC, melanoma, and other immunogenic tumors. I obtained the preliminary data in the Laboratory of Experimental Immunology at the Immunology Frontier Research Center (Osaka University). It was shown that in addition to ROR+Foxp3+ cells eTreg cells are a source of ex-Th17 CD4lowCD25hiCD49hiFoxp3hi (Regulatory Killer T (RKT)) cells while the latest are much more suppressive. Moreover, we have identified a set of key cytokines (low amounts of TGF-*β* and high amounts of IL-2, IL-12, IL-1*β*, and IL-23) that favor the generation and expansion of ex-Th17 Foxp3low cells, although further studies are needed to validate this concept [24].

## Figures and Tables

**Table 1:** General characteristics of patients.

Sex	
Males	15 (83.3%)
Females	3 (16.7%)

Mean age, years	55 (41–77)

Histological variant	
Clear cell RCC	18 (100%)

Prognosis (MSKCC)	
Favorable	11 (61.1%)
Intermediate	7 (38.9%)

Stage IV (at the moment of immunotherapy)	
After palliative nephrectomy	6 (33.3%)
After radical nephrectomy	12 (66.7%)

Previous therapy	
Yes	2 (11.1%)
No	16 (88.9%)

**Table 2:** Reagent sets used for immunoenzymatic assay.

Indicator	Reagent set	Manufacturer	Measurement range
Interleukin 17 (IL-17A)	Human IL-17A Platinum ELISA	eBioscience, USA	0.5–100.0 pg/ml
Transforming growth factor-*β* (TGF-*β*_1_)	TGF-*β*_1_- ELISA	eBioscience, USA	31.250–2000 pg/ml
Erythropoietin (Epo)	Access® EPO-ELISA	Beckman, USA	0.6–750 mU/ml
